# Chinese version of Yoon Critical Thinking Disposition Instrument: validation using classical test theory and Rasch analysis

**DOI:** 10.1186/s12912-023-01519-y

**Published:** 2023-10-06

**Authors:** Mio Leng Au, Yue Yi Li, Lai Kun Tong, Si Chen Wang, Wai I Ng

**Affiliations:** Kiang Wu Nursing College of Macau, Macau SAR, China

**Keywords:** Critical thinking instrument, Chinese version, Reliability, Validity, Nursing

## Abstract

**Background:**

Despite the availability of a wide range of critical thinking instruments, there was no original design for nurses that has been translated into Chinese. However, only instruments designed specifically for the nursing discipline would be reliable. This study aimed to translate, culturally adapt, and validate the Yoon Critical Thinking Disposition Instrument in the Chinese context.

**Methods:**

A four-step translation process was implemented according to Word Health Organization guidelines, which included forward translation, expert panel review, backward translation, and pre-testing. Experts and nursing students participated in testing the validity and reliability of the Chinese version.

**Results:**

The translation of the instrument went smoothly. According to a confirmatory factor analysis, there was an acceptable fit for the seven-factor model. Content validity indices ranged from 0.6 to 1 at item level, and 0.94 at scale level. In addition, there was extremely high internal consistency and test-retest reliability in the translated instrument. There was a good fit for the items with both person and item reliabilities greater than 0.6 and a separation index of 2.19, respectively. The item location was identified from the wright map as not covering person ability, but the scale did not have a gender-related differential item functioning.

**Conclusions:**

In this study, a critical thinking disposition instrument for nursing students was translated into Chinese for the first time. This translated instrument is a reliable tool with satisfactory validity and reliability. It could provide opportunities for building a cross-cultural understanding of critical thinking disposition.

**Supplementary Information:**

The online version contains supplementary material available at 10.1186/s12912-023-01519-y.

## Background

Critical thinking (CT) competence is a cognitive process with attitudinal dispositions (CT disposition) as well as rigorous and autonomous reasoning (CT skill), an indispensable competence in the nursing discipline worldwide [1, 2]. Nurses who maintain CT competence may improve their ability to use reasoning, judgment, and decision-making in the clinical setting [3], and can ensure the safety of patients [4, 5]. Therefore, there is a need for nurses with high levels of CT competence [6]. As a result, CT competence has become an indicator of accreditation standards for nursing education programs [7]. In order to achieve this, the nursing students’ CT needs to be assessed with an effective tool.

Various instruments, including Blooms Taxonomy, California Critical Thinking Disposition Inventory (CCTDI), California Critical Thinking Skills Test, Concept Map Scoring, Critical Thinking Ability Scale, Critical Thinking Assessment, Critical Thinking Disposition Scale, Critical Thinking Process Test, Critical Thinking Scale, Discussion Board Analysis, Health Science Reasoning Test, N3 Case Report Accreditation Form, Think Aloud Analytic Framework, Think Aloud Protocol, and Watson-Glaser Critical Thinking Appraisal, have been used to assess the CT competence of nursing students in both Western and Eastern countries (including China) [8]. With the aforementioned CT instruments, nursing educators might assess CT disposition and CT skill, or both, due to the significant correlation between CT disposition and CT skills in the nursing discipline [9]. Nevertheless, a systematic review concluded that CT instruments that were not designed for the nursing discipline had low reliability, inconsistent reliability, or no reliability reported in nursing education research [8]. Hence, previous research results on CT competence in the nursing discipline need to be questioned. For reliable results of CT competence, Carter et al. [8] pointed out that only the CT instruments designed for the nursing discipline should be utilized by nurses and nursing students. A variety of CT measurement tools have been developed for nursing students from different cultures, such as the Critical Thinking Process Test (CTPT) [10] from Western cultures and the Critical Thinking Disposition Scale for Nursing Students (CTDS) [11] from Eastern cultures. A large number of CT measurement tools developed in accordance with oriental culture originate in South Korea, including CTDS, Critical Thinking Skill Evaluation Scale for Nursing Students [12], Yoon Critical Thinking Disposition Instrument (YCTD) [13]. Nevertheless, the first two scales contain more items. Participants are more likely to complete a survey with a shorter scale, and they will focus more on each question [14]. This highlights the need for an instrument that is short, valid, and reliable. The 27-item YCTD might be an appropriate method to assess nursing students’ CT.

This English version of the YCTD was originally developed to measure CT in Korean nursing students in accordance with oriental culture [15]. It was developed based on the CCTDI [15], the most widely used tool for measuring CT. The subscales of the YCTD are similar to those of the CCTDI, including objectivity, prudence, systematicity, intellectual eagerness/curiosity, intellectual fairness, healthy skepticism, and CT self-confidence [13]. Objectivity in CT refers to the inclination to eliminate personal biases, while prudence entails the habit of recognizing the intricacies inherent in various issues. Furthermore, systematicity involves the inclination to approach problems in a methodical manner, and intellectual eagerness/curiosity denotes the desire to acquire knowledge. Intellectual fairness encompasses the tendency to consider multiple perspectives, while healthy skepticism involves the habit of consistently seeking the most comprehensive understanding of any given situation. Lastly, CT self-confidence pertains to the inclination to rely on reflective thinking in order to resolve problems and make informed decisions. There are 27 items on the YCTD, ranging from 1 (strong disagreement) to 5 (strong agreement), with a higher score indicating a stronger critical thinking tendency [13]. The YCTD has well-established reliability and validity [13].

The YCTD is widely used in different scenarios. Kim et al. employed it to investigate the correlation between nursing students’ personal encounters with incivility and their CT abilities during clinical practice [16]. The results indicated that there was no statistically significant association between the experience of incivility and the scores of the YCTD [16]. The YCTD has also been used by another research team to examined the relationship between academic achievement and CT among nursing students [17]. The results showed that the two variables were positively correlated [17]. The YCTD has also been applied to compare differences in CT among nursing students across nursing programs and academic years [18]. It was observed that the baccalaureate nursing programs students in the higher academic years exhibited a propensity for achieving elevated scores. However, this correlation was not discernible among students enrolled in associate degree programs [18]. In previous studies, the YCTD has been shown to be effective in evaluating the CT of nurse students across programs, academic years, and clinical experiences.

To the best of our knowledge, the CCTDI is frequently utilized among nursing students in China. Considering the length of time it takes to complete the questionnaire and the cost of using it, the 27-item YCTD may be a good choice. Therefore, the purpose of this study was to translate YCTD into Chinese and examine its psychometric properties.

## Methods

### The process of the translation of C-YCTD

First, the original instrument was authorized by the author. In accordance with Word Health Organization (WHO) guidelines [19], four steps need to be implemented: (1) Forward translation - Two bilinguals were invited to independently translate the source language into Chinese. Their native language was Chinese. They studied, worked, and earned PhDs in the U.S. and Canada. The conceptual translation was introduced by one of the authors; (2) Expert panel - A convened expert panel consisting of two translators, five nursing educators, and an English lecturer engaged in a comprehensive discussion regarding the disparities observed between the original text and the two translated version, resulting in the initial Chinese version based on Chinese cultural and grammatical consensus; (3) Backward translation - Two other bilinguals who were unaware of the original instrument were invited to conduct the backward translation (over the past ten years, they have lived in the UK for academic studies after growing up in China. Both completed master’s degrees, and one pursued a PhD in the UK). The same expert panel engaged in a discussion of the back-translation in a manner consistent with the discussion of the forward-translation. The panel conducted a comparative analysis between the original English version and the back-translated version to ensure the preservation of content and conceptual integrity. Subsequent modifications were applied to the adapted forward-translation, resulting in the creation of a pretest Chinese version; (4) Pre-testing and cognitive interviewing – Ten nursing students were invited to participate. Following the completion of the questionnaire, each participant underwent an individual interview to delve into their responses. The purpose of these interviews was to inquire about their comprehension of each item, thereby enabling a comparison between their understanding and the intended meaning of the original scale item. Subsequently, a comprehensive written report was compiled, encompassing all the answers provided by the participants as well as any issues that arose during the interview process. The written report was presented to the same expert panel for discussion. The expert panel engaged in deliberation and consensus-building to address discrepancies, ultimately resulting in the final Chinese version (Appendix 1); 5) Test of the final version – Test-retest reliability was assessed in 31 undergraduate students. A panel of ten experts, comprising nursing and medical experts, and higher education faculty members, was invited to express their opinion on whether the Chinese version of the instrument measures the critical thinking disposition envisioned for content validity and cultural adaptation [20]. The Chinese version of the instrument with an acceptable quality of item-level content validity index (I-CVI) and scale-level content validity index (S-CVI) was sent to participants for further construct validity and reliability testing. Due to the fact that the YCTD was developed on the basis of the CCTDI, and its subscales are also similar to those of the CCTDI, which itself was constructed on the basis of an American Philosophical Association definition of CT disposition, reliability and validity tests were performed using the original seven-factor model [21].

### Participants and settings

In mainland China, 31 provincial-level administrative units are divided into three regions: eastern, central, and western [22]. This cross-sectional study was conducted in the Jiangsu, Hunan, and Sichuan provinces, which were selected to represent the three regions of China.

Students currently enrolled in a college, undergraduate, or graduate nursing program, aged at least 18 years, were included in this study. Students who were unwilling to participate in this study and those who could not read or write in Chinese were excluded. Because factor analysis for developing and refining instruments is recommended to include five to ten participants per item [23], the sample size for assessing construct validity and reliability of the C-YCTD was 300 considering the 27 items on the C-YCTD and loss of participants.

### Procedures and ethical consideration

This study was approved by the Research Management and Development Department of a college of Macau (No. REC-2021.801). The original author granted permission to use and translate the YCTD into Chinese. Each region was assigned a contact person responsible for recruiting eligible participants in their region on social media. The online survey tool Wen Juan Xing was used to collect data. The participants who were willing to join this study had to read the informed consent and click “Agree” button before responding to the survey. In the survey, participants could withdraw at any time and their responses were anonymous. The data collection period was from January 20–26, 2022. The collection and storage of survey data were facilitated by a secure online survey platform, which exclusively permits access to authorized personnel. Access to the survey data is restricted to those individuals who possess the account password and are using designated computers.

### Data analysis

The validity and reliability of the C-YCTD was evaluated in this study. In terms of validity, content validity and construct validity were included, whereas in terms of reliability, internal consistency and test-retest reliability were considered. For content validity, relevance was rated as 1 = not relevant, 2 = somewhat relevant, 3 = quite relevant, and 4 = highly relevant according to the C-YCTD items. The I-CVI was calculated as “the number of experts giving a rating of either 3 or 4 for an item and divided by the total number of experts”; an index greater than 0.7 was considered acceptable [24]. The S-CVI referred to “the summing of the I-CVIs and dividing by the number of items;” in general, greater than 0.8 was considered valid [24]. On the other hand, according to the YCTD based on CCTDI, and the seven-factor model of YCTD proposed strong invariance [25]; the confirmatory factor analysis (CFA) of the seven-factor model provides evidence of construct validity by establishing a model fit with the relationships between items of the C-YCTD using SPSS 26.0 and SmartPLS 4. Furthermore, it is also expected that the results of the CFA should indicate a goodness-of-fit test (χ^2^/df) < 5.0, root mean square error of approximation (RMSEA) < 0.08, comparative fit index (CFI) > 0.90, incremental fit index (IFI) > 0.90 [26], and factor loadings > 0.5 on factors [27, 28]. Convergent validity was assessed by calculating the average variance extracted (AVE), with a threshold of AVE > 0.36 deemed as acceptable [29]. The Heterotrait-monotrait ratio (HTMT) (HTMT < 0.9) was used to examine the discriminant validity [30]. Finally, Cronbach’s α was found to provide satisfactory internal consistency reliability at > 0.7, as well as 0.6 was an acceptable alpha value for a construct containing only four items [31]. The satisfactory intraclass correlation coefficient (ICC) of test-retest analysis was > 0.75 via SPSS 26.0 [24]. A composite reliability (CR) threshold of 0.60 was employed to assess the reliability of factors [32].

The CFA was employed to ascertain the optimal solution for principal component analysis. However, it is important to note that principal component analysis does not yield insights into the distribution and erratic patterns of survey items and respondents, as well as the characteristics of the Likert scale. All Rasch model analysis were performed with Jamovi 2.3 (https://www.jamovi.org). The Wright map was exhibited to provide a comprehensive representation of the distribution of item difficulty and respondent abilities. Infit and outfit mean square (MNSQ) statistics were conducted for both items and respondents in order to detect erratic patterns. MNSQ values falling within the range of 0.6 to 1.4 were considered indicative of optimal fit [33]. Rasch rating scale model reliability statistics for items and persons were computed. Differential item functioning (DIF) is a form of differential validity. It was employed to examine the likelihood of comprehension and response to an item among individuals of varying genders. A significance level of 0.05 was established for DIF, with items deemed biased when p < 0.05 [34].

## Results

### Translation of C-YCTD

Translation of the instrument proceeded smoothly. There were only some differences in the characters between the two translators, but their Chinese expressions were essentially the same. For example, native English experts reported that item Q7 “I treat regular matters like new matters when handling them” differs from the original item “I can take my routine and make it seem new” in the backward translation section. It was finally revised to “I arrange my schedule according to my daily routine and keep a sense of freshness towards it all the time”.

### Characteristics of participants

The construct validity of the C-YCTD was assessed with 401 nursing students, and reliability was assessed with 401 nursing students. The participants’ characteristics are shown in Table 1.

**Table 1:** Participants’ characteristics

Variables	Validity (n = 401)		Reliability (n = 401)	
	n	%	n	%
Gender				
Male	40	10.0	42	10.5
Female	361	90.0	359	89.5
Age				
(mean ± standard deviation)	21.3 ± 3.9		21.2 ± 3.4	
Education				
Associate degree	137	34.2	141	35.2
Undergraduate degree	203	50.6	203	50.6
Postgraduate degree	61	15.2	57	14.2
Regions				
Eastern	151	37.7	151	37.7
Central	120	29.9	120	29.9
Western	130	32.4	130	32.4

### Validity of C-YCTD

The I-CVI ranged from 0.6 to 1 and S-CVI was 0.94. Despite having an I-CVI lower than 0.7, item 7 was retained as a reflection of critical thinking routine scenarios.

**Fig. 1:**
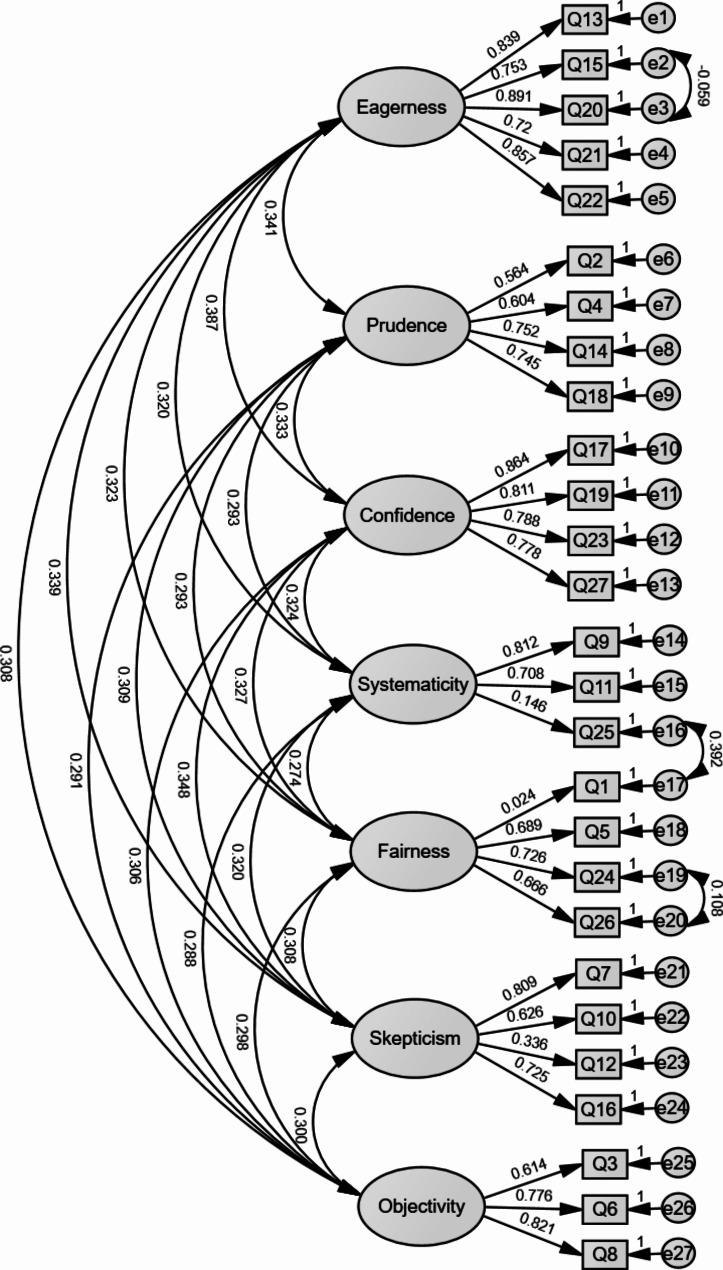
The confirmatory factor analysis of the C-YCTD

CFA was conducted to assess the validity of the seven-factor structure (Fig. 1). The model fit statistics showed χ^2^/df = 3.362, RMSEA = 0.077, CFI = 0.90, and IFI = 0.90, indicating good model fit. The convergent validity results were satisfactory, with AVE values ranging from 0.380 to 0.685 exceeding the threshold (Table 2). All HTMT values in the model were less than the threshold value of 0.90 (Table 2), suggesting that the model exhibits satisfactory discriminant validity.

**Table 2:** The validity of C-YCTD instrument

Factor	Average variance extracted	Heterotrait-monotrait ratio						
		Eagerness	Prudence	Confidence	Systematicity	Fairness	Skepticism	Objectivity
Eagerness	0.685	-^a^						
Prudence	0.380	0.797						
Confidence	0.647	0.891	0.845					
Systematicity	0.588	0.871	0.836	0.846				
Fairness	0.528	0.882	0.734	0.854	0.84			
Skepticism	0.430	0.883	0.899	0.882	0.886	0.825		
Objectivity	0.550	0.831	0.76	0.83	0.859	0.829	0.844	

Note. ^a^Table is symmetric, only the lower triangle is presented

### Reliability of C-YCTD

The reliability of the C-YCTD instrument is shown in Table 3. The overall Cronbach’s α coefficient for the C-YCTD was 0.948, indicating good internal reliability. Cronbach’s α for eagerness, prudence, confidence, systematicity, fairness, skepticism, and objectivity was 0.907, 0.649, 0.877, 0.773, 0.802, 0.676, and 0.779, respectively. The composite reliability for eagerness, prudence, confidence, systematicity, fairness, skepticism, and objectivity was 0.915, 0.640, 0.877, 0.808, 0.810, 0.736, and 0.783, respectively. In addition, excellent test-retest reliability was observed (ICC = 0.963). There were no reported problems in understanding the questions or answering responses during the pretest.

**Table 3:** The reliability of C-YCTD instrument

Factor (Cronbach’s Alpha, Composite reliability) (n = 401)	Item	Score (n = 401)	Cronbach’s Alpha if item deleted (n = 401)	Intraclass correlation coefficient (n = 30)
Eagerness (0.907, 0.915)	Q13	3.71 ± 0.71	0.887	0.893
	Q15	3.61 ± 0.70	0.908	0.811
	Q20	3.79 ± 0.70	0.951	0.879
	Q21	3.77 ± 0.68	0.876	0.908
	Q22	3.64 ± 0.71	0.881	0.859
Prudence (0.649, 0.640)	Q2	3.57 ± 0.73	0.528	0.981
	Q4	3.56 ± 0.73	0.549	0.887
	Q14	3.09 ± 0.73	0.422	0.908
	Q18	3.78 ± 0.67	0.589	0.888
Confidence (0.877, 0.877)	Q17	3.67 ± 0.67	0.834	0.852
	Q19	3.69 ± 0.67	0.837	0.881
	Q23	3.65 ± 0.77	0.854	0.844
	Q27	3.63 ± 0.71	0.844	0.847
Systematicity (0.773, 0.808)	Q9	3.76 ± 0.69	0.678	0.825
	Q11	3.55 ± 0.74	0.694	0.792
	Q25	3.59 ± 0.70	0.711	0.802
Fairness (0.802, 0.810)	Q1	3.82 ± 0.71	0.768	0.965
	Q5	3.62 ± 0.72	0.784	0.935
	Q24	3.82 ± 0.68	0.720	0.800
	Q26	3.76 ± 0.68	0.737	0.866
Skepticism (0.676, 0.736)	Q7	3.58 ± 0.72	0.616	0.897
	Q10	3.37 ± 0.74	0.543	0.921
	Q12	3.25 ± 0.86	0.721	0.833
	Q16	3.55 ± 0.73	0.550	0.947
Objectivity (0.779, 0.783)	Q3	3.79 ± 0.73	0.788	0.943
	Q6	3.77 ± 0.69	0.662	0.890
	Q8	3.86 ± 0.72	0.649	0.835
Overall (0.948)				0.963

### Rasch rating scale model results

**Fig. 2:**
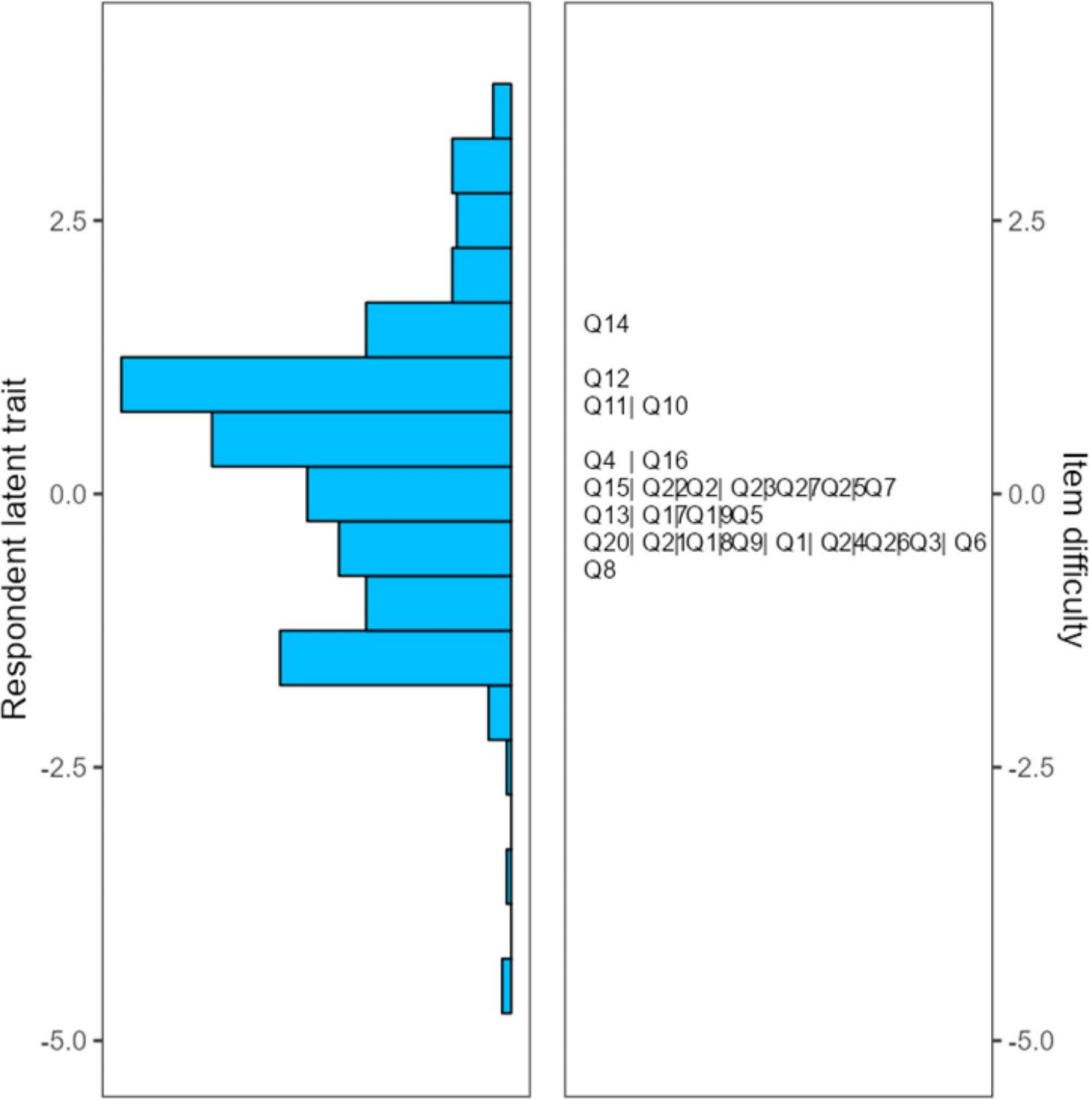
The Wright map of the C-YCTD

Figure 2 provides evidence of comprehensive coverage of ability ranges among nursing students, thereby suggesting the representativeness of the items. The right side of the Fig. 2 illustrates the distribution of items arranged according to their level of difficulty, with the least challenging item (Q8 - “When I don’t agree with the opinion of another person, I explain the reason why I don’t agree”) positioned at the bottom and the most difficult item (Q14 – “When I make a judgement or decision, I rush in making that decision.”) positioned at the top.

The MNSQ indices for both the infit and outfit corresponding to all response categories exhibit a range of values from 0.646 to 1.278, as presented in Table 4. This indicates a satisfactory fit of the Rating Scale Model. The Rasch analysis yielded a person reliability coefficient of 0.945 and a separation index of 2.19, indicating high levels of confidence and discrimination in distinguishing between approximately two levels of individual ability. In terms of reliability assessment, both item reliability (all exceeding 0.60) and person separation reliability (all exceeding 0.60) demonstrated excellent performance across all seven components. Except for item 4 (“When I make a decision, I tend to hurry in reaching a conclusion without consideration.”), there were no significant differences between male and female in their responses to the questionnaire items, and no DIF was observed for the C-YCTD questionnaire items.

**Table 4:** Rasch rating scale model results of the C-YCTD

Item	Infit MBSQ	Outfit MNSQ	Item reliability	Person separation reliability	Differential item functioning	
					Statistic	p
Eagerness			0.86	0.795		
Q13	0.913	0.894			0.412	0.814
Q15	1.076	1.044			0.283	0.868
Q20	0.718	0.646			1.367	0.505
Q21	0.757	0.704			1.378	0.502
Q22	0.815	0.819			0.791	0.673
Prudence			0.638	0.656		
Q2	0.870	0.871			0.560	0.756
Q4	1.271	1.278			19.637	< 0.001
Q14	1.073	1.119			7.555	0.023
Q18	0.787	0.780			2.282	0.320
Confidence			0.824	0.744		
Q17	0.796	0.746			0.090	0.956
Q19	0.784	0.755			0.696	0.706
Q23	1.087	1.046			2.492	0.288
Q27	0.896	0.873			3.331	0.189
Systematicity			0.737	0.871		
Q9	0.916	0.910			2.716	0.257
Q11	1.004	0.998			1.426	0.490
Q25	0.963	0.953			1.950	0.377
Fairness			0.774	0.692		
Q1	1.069	1.049			5.455	0.065
Q5	1.113	1.115			1.551	0.461
Q24	0.847	0.825			2.950	0.229
Q26	0.877	0.869			4.894	0.087
Skepticism			0.689	0.620		
Q7	0.935	0.937			0.793	0.673
Q10	0.851	0.858			1.146	0.564
Q12	1.353	1.369			3.376	0.185
Q16	0.862	0.850			1.139	0.566
Objectivity			0.732	0.678		
Q3	1.092	1.092			4.208	0.122
Q6	0.868	0.882			0.873	0.646
Q8	0.946	0.932			1.946	0.378

## Discussion

The YCTD has been widely used in South Korea to assess the critical thinking of nursing students [35, 36], but the Chinese translation has never been verified. In this study, the YCTD was translated into Chinese through forward translation, backward translation, cultural adaptation, and a pilot study. The C-YCTD was validated using both the classical test theory and the item response theory. The C-YCTD has demonstrated acceptable reliability and validity among Chinese nursing students, suggesting its potential usefulness as a measure for evaluating critical thinking among Chinese nursing students.

Research teams often invest significant effort in maintaining the quality of translations when importing instruments to other languages [37]. Translation was undertaken using the following procedures to ensure quality: (1) the instrument was translated into Chinese in strict compliance with the WHO guidelines and adjusted according to the Chinese context; (2) multiple consensus meetings were held by bilingual experts, nursing education experts, and translators; and (3) two native English speakers with high education evaluated the consistency between the back-translated version and the original English version. It is recommended that the native speaker of the monolingual language of the original instrument ensure semantic equivalence between the two versions [38]. In addition, this could also provide opportunities for building a cross-cultural understanding of CT disposition [39].

The CFA has validated the initial seven-factor structure of the YCTD, demonstrating acceptable model-fit indices. These findings indicate that the C-YCTD is a suitable instrument for implementation within the Chinese cultural context. However, the classical test theory fails to provide comprehensive understanding of the distribution and erratic patterns exhibited by survey items and respondents [40]. To address this limitation, this study employed Rasch model analysis, which falls under the category of item response theory, to thoroughly examine the scale. The Rasch model item separation and reliability of the components indicate the items have good discrimination power, while person separation and reliability coefficients indicate the survey instrument is of good quality. In both CFA and Rasch model analysis, the seven-factor C-YCTD was found to be appropriate.

The Cronbach’s alpha for the C-YCTD (0.948) was higher than that previously reported (0.842), and all C-YCTD dimensions had higher Cronbach’s alphas than the minimum level recommended. Several studies indicate that reliability coefficients exceeding 0.95 may potentially signify redundancy in the measurement of the intended construct within items, whereas other studies propose a threshold of over 0.90 [41]. The reliability coefficients of the subscales of the C-YCTD demonstrate values below 0.90, with the exception of the eagerness subscale. This finding suggests the need for further investigation into potential item deletion within this particular subscale. It is evident from the high ICC (0.963) for test-retest reliability that the C-YCTD is highly reliable in the long run.

The results of this study were strengthened by using a strict validation method. It is important to note that the number and expertise of experts from different professional groups in nursing education ensure that the Chinese version of the instrument is valid [42]. For expert validity evaluation, nursing educators and nurses were not the only expert participants, but also medical and higher education experts proficient in English and Chinese [20]. In addition, this study recruited a large sample (n = 401) to conduct CFA of the C-YCTD [43]. According to the CFA results, the 27 items of the CYCTD loaded on the same factors as the original [25], proving its validity for measuring critical thinking, as well as its potential for use across cultures.

The wright map provides evidence of comprehensive coverage of ability ranges among nursing students, thereby suggesting the representativeness of the items. The aggregate positioning of the items fell below the average value of individual aptitude, rendering it suitable for moderate levels of critical thinking but insufficient for encompassing the abilities of nursing students at both low and high levels. Consequently, the inclusion of easier or more challenging items is necessary to enhance item differentiation and enable effective implementation of the questionnaire among nursing students with varying levels of ability. The utilization of Rasch analysis has yielded significant insights pertaining to the difficulty levels of items, thereby enabling potential enhancements to the tool through scaffolding and the allocation of varying weights to individual items based on their respective degrees of difficulty. Currently, all items in the original scale are scored equally, but future endeavors can be pursued to assign distinct weights to items of varying difficulty.

The identification of DIF holds significance in safeguarding the scale’s validity, as DIF analysis aids in the recognition of items that exhibit bias [44]. Except for item 4 (“When I make a decision, I tend to hurry in reaching a conclusion without consideration.”), there were no significant differences between male and female in their responses to the questionnaire items, and no DIF was observed for the C-YCTD questionnaire items. The significance of DIF in Item 4 may be attributed to the inherent gender imbalance within the nursing profession, resulting in a considerably higher proportion of female participants in this study compared to male participants. To ensure further validation of the DIF of the C-YCTD, it is recommended to enhance the representation of male participants in future research.

This is the first time a questionnaire about critical thinking dispositions for nursing students has been translated into Chinese. In the present study, empirical evidence is presented for the validity and reliability of the C-YCTD instrument as a means of obtaining critical thinking dispositions of nursing students in the Chinese language; thus, it could expand the scope of critical thinking disposition research to populations of Chinese nursing students. It can be used as a tool to assess nursing students’ critical thinking skills and identify their strengths and weaknesses. Nursing educators can then use this information to develop targeted training programs and interventions to help nursing students improve their critical thinking skills. It could also provide opportunities for building a cross-cultural understanding of CT disposition [39, 45]. In the future, researchers could compare critical thinking abilities of nursing students in Chinese contexts with those in other cultures and languages to identify cultural influences.

## Limitation

One limitation of this study is the utilization of the classical back-translation method. Nonetheless, this translation approach is not without its drawbacks, as it may result in translations that closely adhere to the source text. Consequently, it is advisable to explore alternative translation methods, such as the TRAPD, in future research endeavors. Another limitation of this study pertains to the verification of both convergent validity and discriminant validity, which solely assessed the internal validity of the C-YCTD. Originally, the intended examination of criterion validity using the CCTDI was planned. Nevertheless, the execution of this planned analysis was hindered by the significant expenses associated with employing the CCTDI, thereby rendering it unfeasible within the financial limitations of the study. It is therefore important for future studies to confirm the C-YCTD’s external validity. In this study, the validity and reliability of the C-YCTD were tested for the first time, and while the outcomes were acceptable, it is necessary to gradually expand the test to different provinces in China to ensure that a larger sample size fully represents the Chinese population, further verifying the validity and reliability of the C-YCTD. Furthermore, the research sample in this study was nursing students, and clinical nursing professionals could also be used to test the reliability and validity of the C-YCTD in the future. The C-YCTD may also be useful in understanding clinical nurses’ critical thinking disposition.

## Conclusion

Here, the first critical thinking disposition instrument for nursing students was translated into Chinese, and the results indicate that the translated instrument is a valid and reliable tool with acceptable validity and reliability. The present study makes important contributions to ensuring appropriate teaching strategies among Chinese nursing students by measuring and evaluating critical thinking dispositions. It is recommended to conduct additional studies that involve comparing the Chinese version of YCTD with other critical thinking tools. Furthermore, it is advised to further assess the measurement variance of the Chinese YCTD among diverse Chinese populations residing in various regions and among individuals with different characteristics.

### Electronic supplementary material

Below is the link to the electronic supplementary material.

Supplementary Material 1

## Data Availability

The data that support the findings of this study are available from the corresponding author, upon reasonable request.
