# Supplementary material for: Chinese version of Yoon Critical Thinking Disposition Instrument: validation using classical test theory and Rasch analysis
Source: BMC Nurs. 2023 Oct 6;22:362. doi: 10.1186/s12912-023-01519-y (PMC10559418; doi:10.1186/s12912-023-01519-y)
Supplement: Supplementary file 1 — Supplementary Material 1 [file 12912_2023_1519_MOESM1_ESM.docx]

Appendix 1: Yoon Critical Thinking Disposition Instrument

| Item | Strongly Disagree | Disagree | Somewhat Agree | Agree | Strongly Agree |
| --- | --- | --- | --- | --- | --- |
| Q1. If I make an error, I admit it voluntarily. |  |  |  |  |  |
| Q2. Until I find a valid and sufficient reason, I will not make a judgment but instead will think about it. |  |  |  |  |  |
| Q3. I have my own beliefs that come from a reasonable foundation. |  |  |  |  |  |
| Q4. When I make a decision, I tend to hurry in reaching a conclusion without consideration. |  |  |  |  |  |
| Q5. If the truth is revealed, I accept it even if it differs from my point of view. |  |  |  |  |  |
| Q6. I believe that any opinion has to have a valid reason to support it. |  |  |  |  |  |
| Q7. I can take my routine and make it seem new. |  |  |  |  |  |
| Q8. When I don't agree with the opinion of another person, I explain the reason why I don't agree. |  |  |  |  |  |
| Q9. Usually, I'm logical while drawing an inference. |  |  |  |  |  |
| Q10. Sometimes I wonder about the contents in a book. |  |  |  |  |  |
| Q11. People think of me as a logical person. |  |  |  |  |  |
| Q12. Sometimes I question my own beliefs. |  |  |  |  |  |
| Q13. I make strong efforts to learn what I don't know. |  |  |  |  |  |
| Q14. When I make a judgement or decision, I rush in making that decision. |  |  |  |  |  |
| Q15**.** I believe that I am able to handle a difficult task by myself. |  |  |  |  |  |
| Q16. I often wonder about things that other people think are normal. |  |  |  |  |  |
| Q17. I strive to solve a complicated problem. |  |  |  |  |  |
| Q18. I don't decide quickly but I ponder on it. |  |  |  |  |  |
| Q19. When I solve an intricate problem, I use the standards that I established. |  |  |  |  |  |
| Q20. If I have a question, I ask for answers. |  |  |  |  |  |
| Q21. I make an effort to understand how to perform something I don't know well. |  |  |  |  |  |
| Q22. When I discover a subject that I don't comprehend I endeavor until I have an understanding of it. |  |  |  |  |  |
| Q23. If I have to make up my mind about something, I decide by myself I don't depend on others to decide for me. |  |  |  |  |  |
| Q24. I willingly accept criticism from others about my opinion. |  |  |  |  |  |
| Q25. When I have a problem, I systematically apply a problem solving process. |  |  |  |  |  |
| Q26. I evaluate my opinion and the opinion of others fairly. |  |  |  |  |  |
| Q27. I trust my ability to reason when looking for a solution. |  |  |  |  |  |
